# Fabricating a Pickering Stabilizer from Okara Dietary Fibre Particulates by Conjugating with Soy Protein Isolate via Maillard Reaction

**DOI:** 10.3390/foods9020143

**Published:** 2020-01-31

**Authors:** Tolulope Joshua Ashaolu, Guohua Zhao

**Affiliations:** College of Food Science, Southwest University, Chongqing 400715, China; ashaolut@gmail.com

**Keywords:** okara dietary fibre, soy protein isolate, conjugates, Pickering emulsion, micronization, Maillard reaction

## Abstract

Okara is underutilized despite its numerous values explorable in food products. In this study, okara dietary fibre (ODF) was micronized and decorated with soy protein isolate (SPI) through a Maillard reaction by dry heating at 60 °C. The resulting ODF-SPI conjugates were thermally stable, hydrophilic rather than hydrophobic, and exhibited excellent Pickering emulsion stabilization potentials as indicated in their interfacial behaviour, microstructure, droplet sizes, emulsifying activity index (EAI) and emulsion stability index (ESI). In addition, the conjugates’ structure–function relationships, amino acid profile, and emulsifying potentials are indicative of being employed in the formulation of emulsion-based foods or non-edible products.

## 1. Introduction

The production of protein, oil, milk, and tofu from soybean often lead to about 20% (wet basis) soybean residue generation known as okara. Okara is quick to spoil because of its moisture composition and is therefore treated as animal feed, industrial, or agricultural waste [1]. Dehydrated okara is composed of 55% whole fibre, 21% proteins, 13–14% fats and oils, 1.5% ash and 9–10% moisture [2]. Evidence from a recent study showed that okara insoluble polysaccharides (cellulose, hemicellulose and lignin) have emulsifying and rheological properties [3], and its proteins resemble soy protein isolate [4].

There can be protein–polysaccharide complex formation when these molecules interact and thereby adsorb at the oil–water interface to stabilize an emulsion. Karunasawat & Anprung [5] described proteins and polysaccharides as being commonly used in oil-in-water emulsions. Previously, sesame protein [6], flaxseed gum-whey protein isolate conjugates [7], okara polysaccharides-tofu whey conjugates [8], pectin and whey protein isolates [9] were used to synthesize thickeners and emulsion stabilizers. However, the knowledge of particle size plays an important role in determining okara properties and applications, as found in tofu’s fortification with okara particles within the μm range [10]. In addition, the application of okara insoluble polysaccharides in foods such as rheology, textural, and sensorial qualities have been studied, but with little or no knowledge of its hydrophilicity [1].

Emulsions are widely used in the food and pharmaceutical industries to formulate products such as creams, gels, pastes, ointments, and vaccines [11]. A Pickering emulsion is an emulsion that is stabilized by solid particles, which adsorb onto the interface between the two phases. The most distinct difference between Pickering and conventional emulsions is that the former has solid particles on the interface between two stabilizing liquid phases while the latter uses molecular surfactant to stabilize emulsions [12].

The disadvantages of using protein particles to stabilize Pickering emulsions include aggregation and structural instability, while those of polysaccharide-based particles include poor emulsifying performance and surface activity [13]. Therefore, the modification of the particles surface hydrophobicity [14] or coating of the polysaccharides surface with proteins [15] were recommended to subdue these demerits. The coating may be attained by a Maillard reaction, usually via wet or dry heating methods. Oliver et al. [16] recommended the dry heating method for the sake of long-term storage capacity for commercial applications, as well as the ease of handling when compared to the wet heating method.

To the best of our knowledge, the combinatorial effect of micronization and Maillard inducement on okara insoluble polysaccharides and its proteins or soy protein isolate for emulsifying capacity has not been investigated. Therefore, the aim of this study was to micronize and decorate okara dietary fibre (ODF) with soy protein isolate (SPI) via dry heating method of Maillard reaction at 60 °C, for the fabrication of Pickering emulsion, and to investigate changes in their hydrophobicity. The fibre component of okara is higher than that of its protein, thus it was important to supply a conjugating protein counterpart of the same orisgin, in other to achieve this objective. Thence, ODF was glycated with SPI, followed by characterization and Pickering emulsion fabrication. 

## 2. Materials and Methods

### 2.1. Materials

Soy protein isolate was purchased from Shanghai Yien Chemical Technology Co. Ltd., Shanghai (China). Okara was obtained from Tianrun Food Company (Chongqing, China), and was immediately dried using an electric vacuum drying oven (dzf-6050, Heb Biotechnology Co., Ltd, Xi’an, China) at 45 °C for 4–6 h until coarse enough to be powdered. The fat content was found to be 10–12%. Soybean oil was purchased from a local supermarket in Beibei, Chongqing (China). All other chemicals and solvents used in this study were of analytical grade.

### 2.2. ODF Preparation and Micronization

Dried okara was powdered with a high-speed rotary grinder, and was defatted using petroleum ether solvent with a 45 °C boiling temperature in a Soxhlet extractor for 8 h. The method of Ma, Liu, Kwok, & Kwok [17] was followed for protein removal with slight modifications. The defatted okara was dispersed in deionised water using 1:8 *w*/*v* ratio. The mixture was homogenized and stirred at 80 °C for 30 min at the pH of 4.5, and thereafter centrifuged at 4000× *g* for 15 min. The supernatant was removed while the residue was dried, and defined as okara dietary fibre (ODF). Determination of insoluble protein content in the ODF yielded insignificant amounts (less than 1%) using the Kjeldahl method. ODF was micronized with Laser light scattering Mastersizer 3000 (Malvern Instruments Ltd., Worcestershire, UK) using ultra-pure water as the disperse medium and a circulation pump operating at 3000 rpm.

### 2.3. Preparation of ODF-SPI Conjugates via a Maillard Reaction

A mix of ODF and SPI powders in equal amount (1:1 *w*/*w*) was dispersed in deionised water (1:8 *w*/*v*), and was stirred at pH 7.0 for 4 h at room temperature. The homogenized mixture was stored at 4 °C overnight, freeze-dried, and powdered. The ODF-SPI particles were allowed to form conjugates via a Maillard reaction by dry heating at 60 °C. According to the method of [18], a desiccator containing saturated sodium chloride (NaCl) was placed in the oven at 60 °C for 30 min prior to the Maillard reaction, in order to attain an equilibrium temperature and relative humidity. The water activity (a_w_) of the desiccator was determined to be 0.78. The samples were placed in the same desiccator and heated at 60 °C for 3 days (6, 12, 24, 48 and 72 h). Afterwards, the reaction products were collected and defined as conjugates, sealed and stored at 4 °C for further analyses.

### 2.4. Characterization of ODF-SPI Conjugates

#### 2.4.1. Degree of Substitution

OPA (o-phthaldialdehyde) with serine standard was used to determine the degree of substitution (DS) following the method of Nielsen et al. [19]. The DS was calculated as *DS* (%) = (*A*_0_-*A*_t_)/*A*_t_ × 100. The absorbance of each sample before and after Maillard reaction time for t min represent *A*_0_ and *A*_t_, respectively.

#### 2.4.2. Fourier Transform Infrared (FT-IR) Spectroscopy

FT-IR spectra of the conjugates were obtained using a Spectrum 100 FT-IR detector (PerkinElmer, Shelton, CT, USA). Potassium bromide (KBr) discs constituting 3.3% ground conjugates were made, and 32 spectra scans were taken within the range of 400 to 4000 cm^−1^, using a resolution of 4 cm^−1^.

#### 2.4.3. Thermogravimetric Analysis (TGA)

At a heating rate of 10 °C/min, temperature was increased from 35 °C to 600 °C as 8 mg of each sample was analysed for pyrolytic stability under nitrogen protection in a thermogravimetric analyzer 550 (Columbia, TA, USA). Then, the weight loss was measured as a function of temperature. 

#### 2.4.4. Contact Angle Measurement

Measurement of the conjugate wettability was achieved with a goniometer (JC2000D, Jinhua Jianjin Hardware Tools Co., Ltd, Zhejiang, China), after making tablets of each sample by pressure machine. A droplet of water was released unto the surface of the tablet as the drop image was captured and measured as the contact angles based on Laplace-Young model [20].

#### 2.4.5. Amino Acid Composition

The conjugates were hydrolysed at 110 °C for 16 h with 6 M HCl, followed by identification and quantification of their amino acids with respective standards on a HITACHI L-8900 (Hitachi, Tokyo, Japan) HPLC system.

### 2.5. Determination of Emulsifying Properties

#### 2.5.1. Preparation of Pickering Emulsions

At room temperature, 2% salt solution constituting LiOH/KOH/urea/H_2_O (4.5:7:8:80.5 *w*/*w*/*w*/*v*) was used to make conjugates dispersions (5% *w*/*v*), followed by homogenisation with soy oil at 1:2 and 1:3 for 2 min, through a super high-speed shear homogenizer (T25, IKA, Wilmington, NC, USA) at 20,000 rpm.

#### 2.5.2. Emulsifying Activity Index (EAI) and Emulsion Stability Index (ESI)

The absorbance values of freshly prepared emulsions at 0 (*A*_0_) and 30 (*A*_30_) minutes were obtained at 500 nm following the introduction of 25 μL of samples into 5 mL of 0.1% sodium dodecyl sulfate (SDS). Then, emulsifying activity index (EAI) and the emulsion stability index (ESI) were measured and calculated as follows: *EAI* (m^2^/g) = 2 × *T* × *A*_0_ × 200/*c* × *Ø* × 10^4^ and *ESI* (min) = *A*_0_/(*A*_0_ − *A*_30_) × 30.

*T* = 2.303, 200 is dilution factor, *c* is the initial concentration of protein (g/mL), *Ø* refers to oil volume fraction of the emulsion, *A*_0_ and *A*_30_ are the absorbance at 0 and 30 min, respectively.

#### 2.5.3. Colour Analysis

Emulsions colour were recorded at room temperature with a reflective D65 illumination source of a UltraScan Pro (HunterLab, Reston, VA, USA), and expressed in L * (lightness), a * (greenness and redness) and b * (blueness and yellowness) values. The total colour difference (∆E) was calculated with the equation of ∆E = [(L* − L_0_)^2^ +(a − a_0_)^2^ + (b* − b_0_)^2^]^1/2^.

#### 2.5.4. Interfacial Rheology Measurements

Rheological measurements of the conjugate-stabilized emulsions were obtained using an Anton Paar MCR 302 rheometer (Anton Paar, Austria) that was fitted with a 49.978 mm diameter × 1.017 mm thick platinum measuring cone. Two millilitres of emulsions were pipetted onto the plate while lowering the cone was for surface contact. Angular frequency range (γ) of 0.01% to 100% at ω = 1 rad s^−1^ was used for dynamic strain sweeps.

#### 2.5.5. Microstructure Analysis and Droplet Size Measurement

Emulsion droplets were pipetted on microscope glass slides, after which optical microscope (Olympus BX53F, Tokyo, Japan) was used to observe and compare the shapes and microstructure of the ODF-SPI Pickering emulsions. The droplet size distributions were measured using the Image J program, counting at least 50 droplets from different images.

### 2.6. Statistical Analysis

Experiments were repeated in triplicates and the values were expressed as mean ± standard deviation. Significant difference of *p* < 0.05 was taken in a SPSS 22.0 one-way analysis of variance (ANOVA). The data was normally distributed.

## 3. Results and Discussion

### 3.1. Physicochemical Properties of ODF-SPI Conjugates

**Figure 1:**
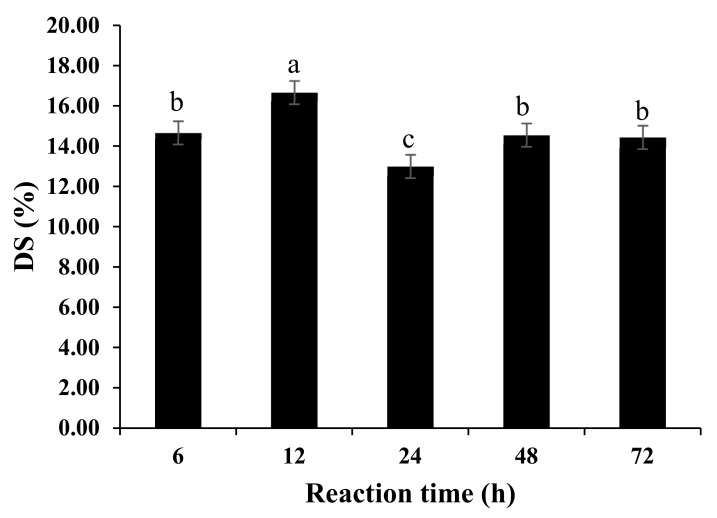
Effect of Maillard reaction time on the degree of substitution in ODF-SPI conjugates. Different letters indicate significant difference.

Maillard reaction can create linkages between the amino groups of proteins and the carbonyl groups of reducing sugars present in a reacting mixture. This reaction can be evaluated by determining the degree of substitution (DS) of the amino groups with the carbonyl groups. The DS of ODF-SPI conjugates as shown in Figure 1 indicated maximum attainment at 12 h, possibly the appropriate glycation time to spatially conform soy protein for the exposure of its active sites of arginine, histidine, lysine, phenylalanine, tyrosine, leucine and asparagine residues as later found (Table 1). A longer time of heating could have caused protein polymerization and reorientation of reactive amino groups, which could be hiding from forming bonds with the carbonyl groups [21]; and formation of Schiff base could have led to loss of some active amino groups [22]. Moreover, the Maillard reaction itself results in several intermediates and glycation products, which are affected by many factors [21,23]. In the same vein, carbohydrate molecules are prone to readily react with lysyl residues that could eventually result in insoluble aggregates due to the progressive side reaction [24,25,26].

All conjugates exhibited substitution degrees within 13–17% range, after using an equal amount of SPI and okara dietary fibre. Therefore, equal w/w ratios used could not guarantee the availability of same readily available carbonyl and amino groups that could graft each other. Another noteworthy observation was that okara fibre used in this work consisted mainly of high molecular weight insoluble polysaccharides (cellulose, hemicellulose and lignin among others), and might have led to a slower rate of glycation. This concords with the works of Li et al. [27] and Li et al. [26]. 

To ensure that conjugation occurred between soy protein and okara fibre, the amino acid composition was determined. As shown in Table 1, there were major losses in asparagine, histidine, lysine, isoleucine, leucine, tyrosine, phenylalanine, tryptophan and arginine as compared to SPI or ODF-SPI mixture. Loss of arginine and lysine have been associated with conjugation, and the profile obtained in this work is similar to that of soy protein-dextran conjugates obtained in the study of Li and colleagues [26]. The amino groups losses might be due to the overlapping interactions of the intermolecular and intramolecular structures of the ODF and SPI. In contrast, the amino acid contents of SPI remained stable, possibly due to lack of interactions with any external molecule.

**Figure 2:**
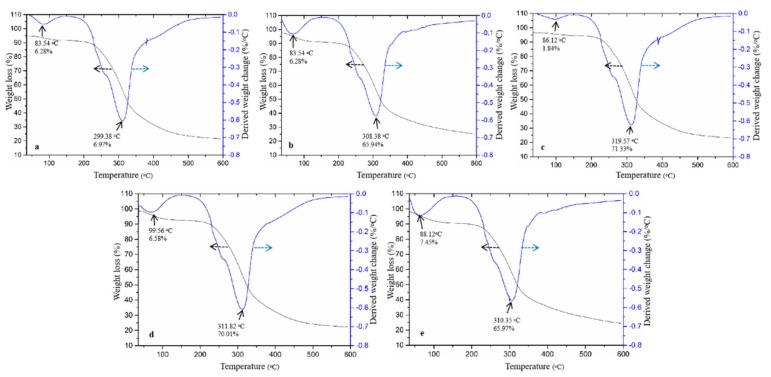
Thermograms of ODF-SPI mixture (**a**) and ODF-SPI conjugates after dry heating at 60 ^o^C for 6 h (**b**), 12 h (**c**), 24 h (**d**) and 48 h (**e**).

Furthermore, the irregularity in the order of increase or decrease of amino acid contents with reaction time might be due to instability or switching of the reacting ε-amino groups of the amino acids with the carbonyl groups during the dry heating process. Other reports have also asserted that conjugation occurred at the tryptophan indole group, arginine guanidine group and all the proteins *N*-terminus in the conjugates [18,28]. Li et al. [26] who used soy proteins and dextran for conjugate formation found lower and varying contents of amino acids as compared to this present study. The conjugates were further analysed for thermal stability, FT-IR spectroscopy and hydrophobicity. Thermograms obtained in Figure 2 showed that the thermal decomposition of conjugates begun from 83.54 °C until the maximum of 319.57 °C.

Onset weight losses as shown by the endothermic peaks, are due to bound water removal from the conjugates. Emergence of the second peaks showed that the conjugates contained some matter that were being oxidized [18], or further decomposition of sugars and amino acids into flammable gases such as carbon monoxide, carbon dioxide and ammonia. The highest weight losses around 300 °C and above suggest that both intra- and inter-molecular hydrogen and electrostatic bonds became broken, leading to the loss of hydrophobic interaction, and followed by the bond breaking of C-N, C(O)-NH, and C(O)-NH_2_ of amino acids with the increase in temperature [29]. This kind of interaction seemed stronger in conjugates obtained after dry heating at 60 °C for 12 h (Figure 2c), which had a decomposition temperature as high as 319.57 °C, thus exhibiting a more thermal stability than other conjugates (Figure 2a–e). The data show that thermal gravimetrics of macromolecules can be very useful to analyse thermal stability of conjugates under continuous heating. This is due to water losses among ODF-SPI conjugates, which indicated varying degrees of hydrogen bonding at higher temperatures. Moreover, based on the conjugates’ residual contents, increase in their percentage weight losses could be associated with increased protein-protein cross-linking interactions during Maillard reaction, thereby leading to higher thermal stability [18].

**Figure 3:**
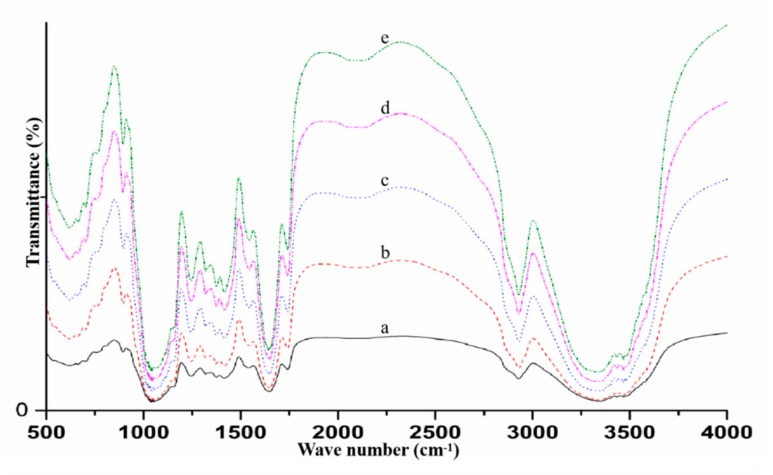
FT-IR spectra of ODF-SPI mixture (**a**) and ODF-SPI conjugates after dry heating at 60 °C for 6 h (**b**), 12 h (**c**), 24 h (**d**) and 48 h (**e**).

Fourier transform infrared spectroscopy is also useful when observing the characteristics of Maillard induced conjugates [30], such as elucidation of their structures and interactions. The adsorbed spectra bands are matched with amide A and amide B, to understand hydrogen bond formation and peptide chains during N-H and O-H, and amino groups interactions, respectively [20]. Amide I, II, and III are employed to study the Maillard reaction (C=O stretching, N-H bending, C-N and N-H bending vibrations, respectively) between polysaccharides and proteins [18]. The spectra of ODF-SPI mixture and conjugates as shown in Figure 3 depict the infrared bands to have been absorbed at 1015 cm^−1^, 1650 cm^−1^, 2925 cm^−1^, and 3424 cm^−1^ regions. In comparison with the ODF-SPI mixture (a), and based on consistency, increasing minor shifts in peaks according to the reaction time of the conjugates (b–e) showed that Maillard inducement by dry heating at 60 °C affected the interaction of hydrogen bonds and their covalency. The intensity of all the bands around the 1015 cm^−1^ region demonstrated that the polysaccharides bound to the peptides, when matched with C-O and C-N stretching vibrations [26]. Also, the absorption of conjugates at 2925 and 3424 cm^−1^ regions, can be associated with -C-H (SP^3^) antisymmetric and -O-H stretching vibrations, respectively [26].

The presence of amide I band (1630–1650 ·cm^−1^) consistent with the conjugates within its region, suggest Schiff’s base imine and enaminol groups (stretching) [31] to have been formed during the heating process. Overall observations showed that covalent bonds between the -NH_2_ and carbonyl groups occurred.

**Figure 4:**
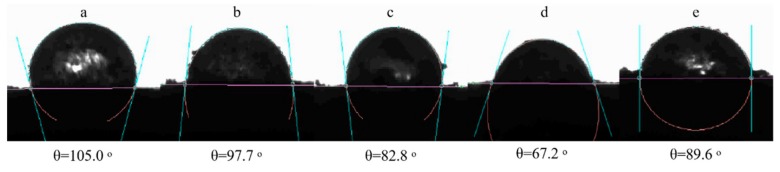
Contact angle measurement of ODF-SPI mixture (**a**) and ODF-SPI conjugates after dry heating at 60 °C for 6 h (**b**), 12 h (**c**), 24 h (**d**) and 48 h (**e**).

Determination of wettability by measuring water contact angles of Pickering conjugates assists in shedding more insights into their surface hydrophobicity [20]. Water droplet contact angles for hydrophilic and highly hydrophilic particles are below 90°and 50°, respectively, while the angles for hydrophobic and highly hydrophobic particles are larger than 90°and 130°, respectively [32]. From Figure 4a–e, ODF-SPI conjugates were hydrophilic except that obtained at 6 h (θ = 97.7°) (Figure 4b) and ODF-SPI mixture (θ = 105°) (Figure 4a). The surface wetting behaviour interferes with the conformational orientation of adsorbed proteins during conjugation, and the range of angles recorded explain how medium or high amount of water vapour may be required to permeate the conjugates.

The protein in the ODF-SPI conjugates might have undergone structural changes induced by heat treatment during dry heating at 60 °C with increase in reaction time. This might have exposed the active amino groups to readily form strong bonds with the carbonyl groups of the ODF, and therefore led to increasing hydrophilicity as compared to the mixtures. These hydrophilic changes can be related to emulsion properties, which may be reduced or increased by the attachment of polysaccharides [18].

### 3.2. Pickering Capacity of ODF-SPI Conjugates

**Figure 5:**
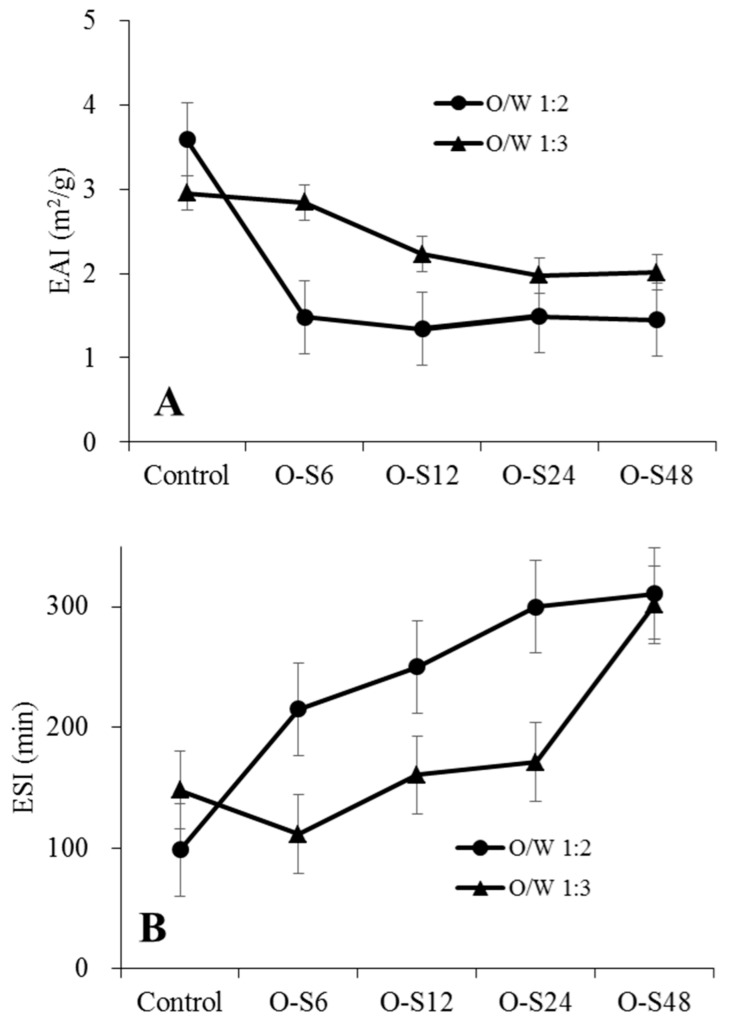
Emulsifying activity index (EAI) (**A**) and emulsion stability index (ESI) (**B**) of Pickering emulsions stabilized by ODF-SPI mixture (control) and ODF-SPI conjugates by dry heating at 60 °C for 6 h (O-S6), 12 h (O-S12), 24 h (O-S24) and 48 h (O-S48).

Emulsions are complex two-phase systems, made by droplets dispersed in a continuous phase [33]. The emulsifying activity index (EAI) and emulsion stability index (ESI) of freshly prepared emulsions stabilized with ODF-SPI conjugates were determined and represented in Figure 5. Different oil/water ratios and reaction times portray different EAI and ESI values. Emulsions prepared with 1:2 O/W had less overall activity (Figure 5A), but better stability than those of 1:3 (Figure 5B), possibly due to the higher concentration of conjugates present in the emulsion system. Higher oil/water ratio improved the emulsion activity for O-S6 (Figure 5A) but reduce stability for all conjugates (Figure 5B).

This could imply that the ODF (insoluble polysaccharides) provided steric stability, and that an increase in O/W ratio would reduce emulsion stability since lower concentrations of particles were available to stabilize the emulsion. For both O/W ratios used, increase in reaction time was directly proportional to increase in stability, which could be due to formation of stronger bonds within the conjugates as more reducing sugars and amino groups were made available with increased glycation time during Maillard reaction. The emulsifying properties were significantly enhanced by polysaccharides through steric stabilization [18], and the presence of hydrophilic molecules. Previous studies on the emulsifying properties of okara polysaccharides, tofu whey conjugates [8], and okara polysaccharides to produce thickeners and emulsion stabilizers [34] are in accordance with this study. Several proteins are usually large and folded, possessing amphiphilic properties that are good for interfacial adsorption at multiple contact points [8]. Unfolding or denaturation of proteins using methods such as the Maillard reaction may expose the modified proteins to other contact molecules present in the emulsion system such as polysaccharides, which then improves their stabilisation and formation of Pickering emulsions.

**Figure 6:**
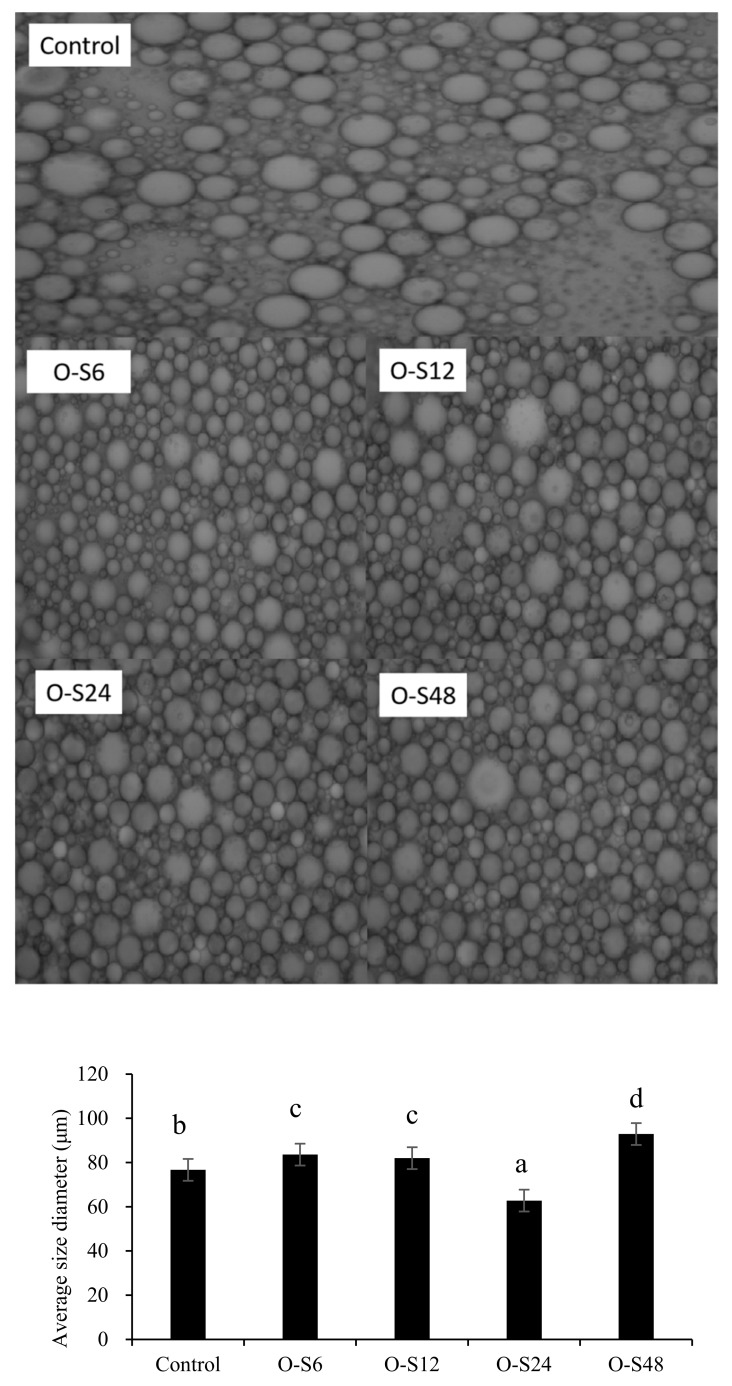
Microstructure of freshly prepared emulsions fabricated with ODF-SPI mixture (control) and ODF-SPI conjugates by dry heating for 6 h (O-S6), 12 h (O-S12), 24 h (O-S24) and 48 h (O-S48), and their average droplet sizes using 1:2 oil/water ratio. Significant differences compared with the Control at *p* < 0.05 level were denoted by the superscripts.

Based on stabilizing potentials, Pickering emulsions prepared with 1:2 O/W were further employed for microstructure and droplet size analyses. The micrographs shown in Figure 6 indicate that the oil droplets have spherical shapes bound by the conjugates. The average droplet size diameter (μm) showed that the droplets are less than 100 μm and might have justified why the emulsions had good emulsifying potentials (Figure 5). Smaller particles require less mass to capture an interfacial area than larger ones for Pickering emulsion stabilization, thus interfacial mechanisms favour smaller particles than larger ones. The increased oil/water ratio (1:3) from Figure 5, had less emulsion stability effect and flocculation possibly due to the decreased number of particles available to be transported into the oil droplets (data not shown). The coating of the lipid droplets by ODF-SPI conjugates and presence of many droplets suggest thick adsorption layer formation at the emulsion interface that can increase steric repulsion [35], which may lead to mixed or multi-layer structures [36].

The thick adsorption layer may prevent the particles from aggregating the lipid droplets and thus increase the stability of emulsions as demonstrated by all the ESI values (Figure 5B).

### 3.3. Interfacial Rheological Behaviours of Pickering Emulsions

**Figure 7:**
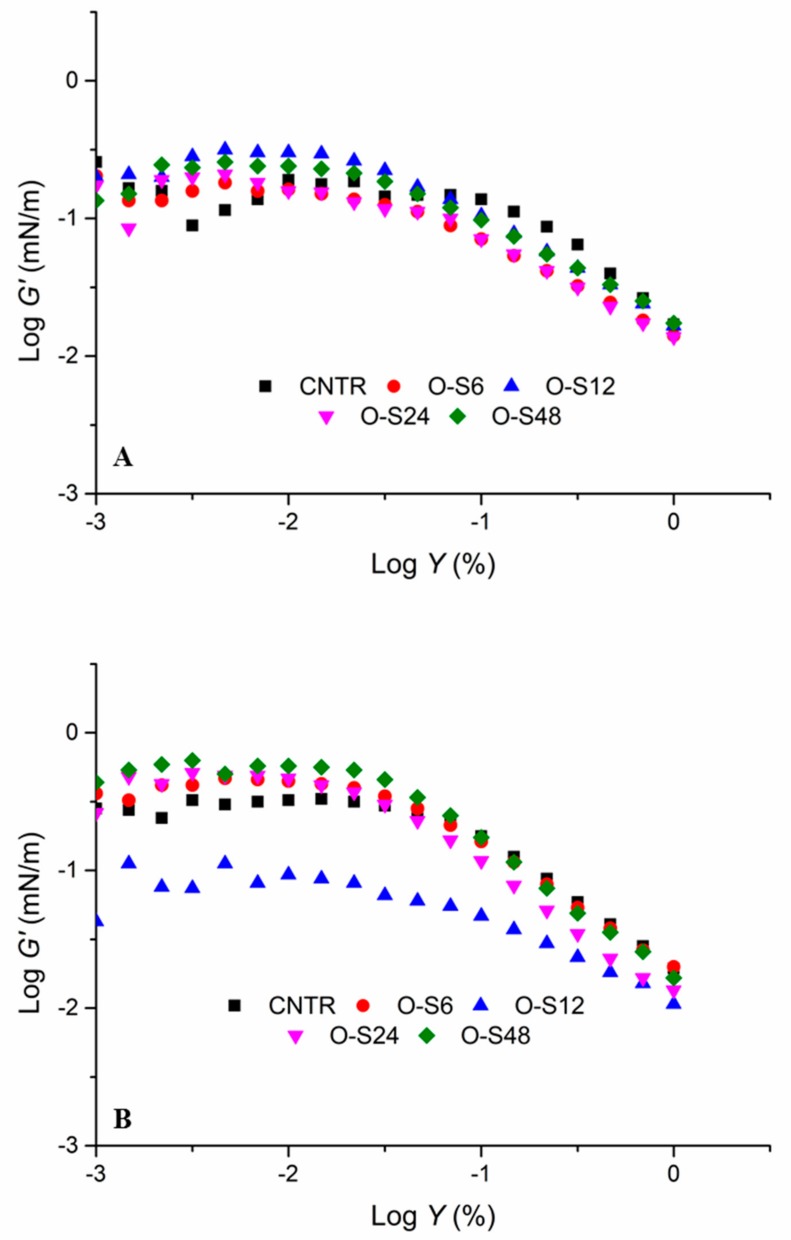
Strain dependence of interfacial storage modulus (*G*′) of Pickering emulsions stabilized by ODF-SPI mixture (control = CNTR) and ODF-SPI conjugates by dry heating for 6 h (O-S6), 12 h (O-S12), 24 h (O-S24) and 48 h (O-S48) with oil/water ratios of 1:2 (**A**) and 1:3 (**B**).

The surface shear rheology of the Pickering emulsions was analysed because it is important for the study of polysaccharide–protein complexes in emulsions. Surface shear rheology is an important physical behavioural measurement that can show how materials deform or flow in response to applied forces or stresses. It involves the flow of liquids and the deformation of solids. Surface active proteins dominate the primary layers around the droplets while reacting polysaccharides form the outer secondary stabilizing layers [37]. The interfacial storage moduli (G′) dependent on strain for the ODF-SPI fabricated emulsions are shown in Figure 7. The strain sweep demonstrated gradual diminishing in G′ values irrespective of glycation time, indicative of the viscoelastic as well as emulsifying effects of the conjugates. It implies that the ODF-SPI conjugates showed very strong intermolecular forces [20], connoting stronger bond formations when prepared as emulsions. The gradual breakage of interfacial films signified the possibility of the conjugates to maintain intact structures after deformation in emulsion systems. This pattern concords with the interfacial rheology measurements of film-forming silk fibroin [38] and soy protein-acacia gum complexed emulsion-based films using different essential oil types [20]. Conjugates prepared at 12 h (O-S12) had a better adsorption and desorption performance at the interface of the oil–conjugate complex layer, in O/W 1:2 (Figure 7A). This might be due to the decreasing hydrophobic groups present after glycation [18], creating a haphazard weakening rate of the interfacial film.

### 3.4. Colour Parameters of Pickering Emulsions

The Maillard reaction involves a series of reactions that significantly contribute to consumers’ acceptability of foods, apart from affecting the structure, flavor, and colour of food products [4]. Other than that, the colour parameter is used for measuring the degree of reaction of Maillard products and the quality of food ingredients. The colour values (L *, a *, b *) of emulsions fabricated with ODF-SPI conjugates are presented in Table 2. In general, emulsions prepared with 1:2 O/W exhibited greater lightness and yellowness (L * and b *) values than those of 1:3 O/W. These values were possibly affected by the concentration of conjugates (5% *w*/*v*) and O/W ratios (1:2, 1:3) used in fabricating the emulsions. They also possessed some redness (a *) values in significant proportions as compared to the control. These values suggest that the conjugates might have undergone certain reactions, such as condensation and polymerisation, which ultimately led to the non-enzymatic Maillard browning. The colour representations suggest attractiveness to consumers or users of formulated colourful food and non-edible products such as creams and ointments.

## 4. Conclusions

Decoration of micronized okara dietary fibre with soy protein by dry heating method of Maillard reaction portends emulsifying potentials. Thermal stability, wettability, and structure–function characteristics of the ODF-SPI conjugates showed that they may serve as Pickering stabilizers, as demonstrated by their EAI and ESI, microstructure, droplet sizes, and interfacial rheological behaviours. The demerit of this study is the minute amount (2%) of salts used in preparing the Pickering emulsions, which is not so suitable for food but for non-edible applications. It is supposed that the pharmaceutical and food industries could find the conjugates useful in formulating emulsion-based products. Thus, future studies will include the elimination of salts, assessment of physicochemical parameters, safety, and health prospects of the conjugates.

## Figures and Tables

**Table 1:** Effects of micronization and Maillard reaction on the amino acid profiles of ODF-SPI conjugates.

Amino Acid (%, Dry Weight Basis)	SPI	ODF-SPI Mixture	O-S6	O-S12	O-S24	O-S48
Aspartic acid (Asp)	0.86	0.71	0.66	0.58	0.64	0.59
Threonine (Thr)	0.25	0.19	0.52	0.47	0.51	0.40
Serine (Ser)	0.30	0.23	0.89	0.81	0.89	0.72
Glutamate (Glu)	1.78	1.48	2.41	2.12	2.35	1.97
Glycine (Gly)	0.60	0.43	5.42	4.80	5.92	4.70
Alanine (Ala)	0.55	0.71	0.79	0.67	0.73	1.05
Cysteine (Cys)	0.09	0.10	23.19	23.95	25.06	22.45
Valine (Val)	0.05	0.08	18.81	18.66	12.81	19.05
Methionine (Met)	2.70	2.23	2.89	2.95	3.23	2.64
Isoleucine (Ile)	1.01	3.03	1.88	1.80	1.71	2.02
Leucine (Leu)	12.10	24.25	6.82	6.51	7.31	9.07
Tyrosine (Tyr)	7.95	7.30	3.54	3.56	3.94	2.89
Phenylalanine (Phe)	11.77	10.03	5.17	5.08	5.67	6.01
Lysine (Lys)	11.26	8.54	4.88	4.83	5.36	4.53
Tryptophan (Trp)	28.57	22.13	13.31	14.31	14.05	14.35
Histidine (His)	5.08	4.13	2.19	2.17	2.41	1.94
Arginine (Arg)	14.99	14.06	6.44	6.40	7.09	5.13
Proline (Pro)	0.10	0.15	0.21	0.33	0.31	0.50
Total	100.00	100.00	100.00	100.00	100.00	100.00

Amino acid contents of ODF-SPI conjugates after a Maillard reaction by dry heating at 60 °C for 6 (O-S6), 12 (O-S12), 24 (O-S24) and 48 (O-S48) hours compared with ODF-SPI mixture and soy protein isolate (SPI).

**Table 2:** Colour parameters of Pickering emulsions stabilized by Maillard induced ODF-SPI conjugates.

Sample	L *		a *		b *		∆E	
	1:2	1:3	1:2	1:3	1:2	1:3	1:2	1:3
O-S	67.80 ± 1.58 ^d^	62.49 ± 4.91 ^d^	2.01 ± 0.10 ^b^	1.97 ± 0.62 ^a^	15.66 ± 1.20 ^a^	17.63 ± 1.00 ^b^	-	-
O-S6	58.16 ± 3.12 ^b^	57.62 ± 0.20 ^c^	2.18 ± 0.20 ^c^	2.21 ± 0.09 ^c^	17.72 ± 0.22 ^b^	18.70 ± 0.24 ^d^	9.86 ± 0.51 ^b^	4.98 ± 0.11 ^a^
O-S12	57.61 ± 0.11 ^a^	53.19 ± 3.33 ^b^	2.01 ± 0.50 ^a^	2.38 ± 0.49 ^c^	18.00 ± 1.40 ^c^	18.20 ± 1.00 ^d^	10.46 ± 0.20 ^d^	9.32 ± 0.78 ^c^
O-S24	58.08 ± 3.80 ^b^	54.62 ± 5.00 ^b^	1.79 ± 0.13 ^a^	1.99 ± 0.52 ^b^	18.37 ± 0.33 ^c^	17.89 ± 0.00 ^b^	10.09 ± 0.73 ^c^	7.87 ± 0.45 ^b^
O-S48	59.87 ± 1.21	51.33 ± 3.12 ^a^	2.23 ± 0.31 ^d^	2.83 ± 0.11 ^d^	18.48 ± 0.33 ^c^	16.88 ± 0.51 ^a^	8.42 ± 0.44 ^a^	11.22 ± 0.88 ^d^

Lightness (L *), redness/greenness (a *), yellowness/blueness (b *) and total colour differences (ΔE) of Pickering emulsions stabilized with ODF-SPI mixture (O-S) and ODF-SPI conjugates obtained after dry heating at 60 °C for 6 h, 12 h, 24 h and 48 h (O-S6, O-S12, O-S24 and O-S48, respectively). Significant differences within the same columns at *p* < 0.05 level were denoted by the superscripts.
